# Supplementary material for: Association of plasma aflatoxin with persistent detection of oncogenic human papillomaviruses in cervical samples from Kenyan women enrolled in a longitudinal study
Source: BMC Infect Dis. 2023 Jun 6;23:377. doi: 10.1186/s12879-023-08323-8 (PMC10242809; doi:10.1186/s12879-023-08323-8)
Supplement: Supplementary file 1 — Additional file 1: Supplementary Table 1. Frequency of HPV detections among women at the enrollment, 12-month and 24-month visits. [file 12879_2023_8323_MOESM1_ESM.docx]

Supplementary Table 1. Frequency of HPV detections among women at the enrollment, 12-month and 24-month visits

| HPV | Enrollment N=66 | 12-month visit N=54 | 24-month visit N=61 |
| --- | --- | --- | --- |
| HPV 16 | 2 (3.0%) | 2 (3.7%) | 2 (3.3%) |
| HPV 18 | 1 (1.5%) | 4 (7.4%) | 3 (4.9%) |
| HPV 26 | 0 | 1 (1.9%) | 0 |
| HPV 31 | 0 | 1 (1.9%) | 1 (1.6%) |
| HPV 33 | 0 | 0 | 0 |
| HPV 35 | 1 (1.5%) | 1 (1.9%) | 1 (1.6%) |
| HPV 39 | 1 (1.5%) | 0 | 0 |
| HPV 45 | 1 (1.5%) | 1 (1.9%) | 1 (1.6%) |
| HPV 51 | 0 | 3 (5.6%) | 1 (1.6%) |
| HPV 52 | 4 (6.1%) | 2 (3.7%) | 4 (6.6%) |
| HPV 53 | 3 (4.6%) | 3 (5.6%) |  |
| HPV 56 | 0 | 1 (1.9%) | 2 (3.3%) |
| HPV 58 | 4 (6.1%) | 3 (5.6%) | 1 (1.6%) |
| HPV 59 | 2 (3.0%) | 1 (1.9%) | 1 (1.6%) |
| HPV 66 | 0 | 0 | 0 |
| HPV 67 | 0 | 0 | 0 |
| HPV 68 | 0 | 1 (1.9%) | 2 (3.3%) |
| HPV 69 | 0 | 0 | 1 (1.6%) |
| HPV70 | 2 (3.0%) | 3 (5.6%) | 1 (1.6%) |
| HPV 73 | 0 | 1 (1.9%) | 1 (1.6%) |
| HPV 82 | 0 | 0 | 1 (1.6%) |
| HPV IS39 | 0 | 1 (1.9%) | 0 |
| HPV 6 | 1 (1.5%) | 0 | 1 (1.6%) |
| HPV 11 | 0 | 0 | 0 |
| HPV 40 | 0 | 0 | 1 (1.6%) |
| HPV 42 | 0 | 0 | 1 (1.6%) |
| HPV54 | 1 (1.5%) | 1 (1.9%) | 1 (1.6%) |
| HPV 55 | 1 (1.5%) | 2 (3.7%) | 0 |
| HPV 61 | 1 (1.5%) | 1 (1.9%) | 0 |
| HPV 62 | 1 (1.5%) | 3 (5.6%) | 1 (1.6%) |
| HPV 64 | 0 | 0 | 0 |
| HPV 71 | 0 | 0 | 0 |
| HPV 72 | 0 | 0 | 0 |
| HPV 81 | 1 (1.5%) | 0 | 0 |
| HPV 83 | 1 (1.5%) | 3 (5.6%) | 0 |
| HPV 84 | 1 (1.5%) | 3 (5.6%) | 0 |
| HPV CP6108 | 4 (6.1%) | 0 | 1 (1.6%) |
| Any HPV^1^ | 23 (34.8%) | 24 (44.4%) | 21 (34.4%) |
| HR-HPV^2^ | 18 (27.3%) | 19 (35.2%) | 18 (29.5%) |
| IARC HR-HPV^3^ | 14 (21.2%) | 16 (29.6%) | 15 (24.6%) |
| A9 HPV^4^ | 11 (16.7%) | 9 (16.7%) | 8 (13.1%) |
| Non-HPV 16 A9^5^ | 9 (13.6%) | 7 (13.0%) | 7 (11.5%) |
| A7 HPV^6^ | 4 (6.1%) | 6 (11.1%) | 7 (11.5%) |
| Non-HPV 18 A7^7^ | 3 (4.5%) | 2 (3.7%) | 4 (6.6%) |
| Vaccine-protected HR-HPV^8^ | 12 (18.2%) | 12 (22.2%) | 11 (18.0%) |
| Vaccine-unprotected HR-HPV^9^ | 7 (10.6%) | 11 (20.4%) | 8 (13.1%) |
| LR-HPV^10^ | 8 (12.1%) | 12 (22.2%) | 6 (9.8%) |

^1^Any HPV: HPV 6, 11, 16, 18, 26, 31, 33, 35, 39, 40, 42, 45, 51, 52, 53, 54, 55, 56, 58, 59, 61, 62, 64, 66, 67, 68, 70, 71, 72, 73, 81, 82, 83, 84, CP6108, IS39

^2^HR-HPV (High-Risk HPV): HPV 16, 18, 26, 31, 33, 35, 39, 45, 51, 52, 53, 56, 58, 59, 66, 67, 68, 69, 70, 73, 82, IS39

^3^IARC HR-HPV: HPV 16, 18, 31, 33, 35, 39, 45, 51, 52, 56, 58, 59, 66

^4^A9 HPV: HPV 16, 31, 33, 35, 52, 58

^5^Non-HPV 16 A9: HPV 31, 33, 35, 52, 58

^6^A7 HPV: HPV 18, 39, 45, 59, 68

^7^Non-HPV 18 A7: HPV 39, 45, 59, 68

^8^Vaccine-protected HR-HPV: HPV 16, 18, 31, 33, 45, 52, 58

^9^Vaccine-unprotected HR-HPV: HPV 26, 35, 39, 51, 53, 56, 59, 66, 67, 68, 69, 70, 73, 82, IS39

^10^LR-HPV (Low-Risk HPV): HPV 6, 11, 40, 42, 54, 55, 61, 62, 64, 71, 72, 81, 83, 84, CP6108
