# Supplementary material for: Association of plasma aflatoxin with persistent detection of oncogenic human papillomaviruses in cervical samples from Kenyan women enrolled in a longitudinal study
Source: BMC Infect Dis. 2023 Jun 6;23:377. doi: 10.1186/s12879-023-08323-8 (PMC10242809; doi:10.1186/s12879-023-08323-8)
Supplement: Supplementary file 2 — Additional file 2: Supplemental Table 2. Ordinal logistic regression analyses of IARC HR-HPV, A9 HPV, Non-HPV 16 A9, A7 HPV, Non-HPV 18 A7, and LR-HPV detection (persistent detection vs. incidence detection vs. no detection) with plasma AFB1-lys detection, and demographic/behavioral characteristics of women. [file 12879_2023_8323_MOESM2_ESM.docx]

Supplemental Table 2. Ordinal logistic regression analyses of IARC HR-HPV, A9 HPV, Non-HPV 16 A9, A7 HPV, Non-HPV 18 A7, and LR-HPV detection (persistent detection vs. incidence detection vs. no detection) with plasma AFB1-lys detection, and demographic/behavioral characteristics of women.

| Variables | IARC HR-HPV^1^ | | A9 HPV^2^ | | Non-HPV 16 A9^3^ | |
| --- | --- | --- | --- | --- | --- | --- |
|  | OR (95% CI) | P-value | OR (95% CI) | P-value | OR (95% CI) | P-value |
| Plasma AFB1-lys detection | 2.12 (0.75 - 6.01) | 0.155 | 1.67 (0.55 - 5.13) | 0.367 | 1.99 (0.59 - 6.72) | 0.265 |
| Age | 0.90 (0.81 - 1.01) | 0.064 | 0.93 (0.83 - 1.05) | 0.246 | 0.98 (0.87 - 1.11) | 0.786 |
| Married | 0.31 (0.09 - 1.04) | 0.058 | 0.38 (0.10 - 1.40) | 0.147 | 0.40 (0.11 - 1.51) | 0.176 |
| More than secondary school education | 1.00 (0.24 - 4.25) | 0.999 | 1.86 (0.42 - 8.35) | 0.416 | 3.60 (0.80 - 16.23) | 0.095 |
| Home ownership | 1.99 (0.57 - 6.92) | 0.278 | 0.62 (0.15 - 2.60) | 0.517 | 0.73 (0.17 - 3.20) | 0.676 |
| Walking distance to health care ≥60 mins | 0.42 (0.08 - 2.24) | 0.308 | 0.34 (0.05 - 2.57) | 0.299 | 0.60 (0.08 - 4.40) | 0.617 |
| Number of lifetime sex partners | 1.21 (0.96 - 1.51) | 0.104 | 0.96 (0.74 - 1.25) | 0.751 | 0.94 (0.72 - 1.24) | 0.670 |
| Age of first sex | 1.20 (1.01 - 1.44) | 0.038 | 1.14 (0.96 - 1.37) | 0.140 | 1.01 (0.84 - 1.23) | 0.887 |
| Variables | A7 HPV^4^ | | Non-HPV 18 A7^5^ | | LR-HPV^6^ | |
|  | OR (95% CI) | P-value | OR (95% CI) | P-value | OR (95% CI) | P-value |
| Plasma AFB1-lys detection | 1.06 (0.24 - 4.63) | 0.937 | 0.38 (0.07 - 2.21) | 0.284 | 0.74 (0.20 - 2.66) | 0.642 |
| Age | 0.97 (0.84 - 1.12) | 0.693 | 1.00 (0.85 - 1.18) | 0.983 | 0.85 (0.74 - 0.98) | 0.025 |
| Married | 1.04 (0.18 - 5.95) | 0.968 | 2.21 (0.24 - 20.16) | 0.483 | 0.20 (0.05 - 0.88) | 0.033 |
| More than secondary school education | 2.05 (0.32 - 13.11) | 0.448 | 2.88 (0.33 - 25.42) | 0.340 | 13.35 (2.08 - 85.64) | 0.006 |
| Home ownership | 2.77 (0.49 - 15.56) | 0.248 | 1.01 (0.13 - 7.70) | 0.993 | 2.02 (0.42 - 9.74) | 0.383 |
| Walking distance to health care ≥60 mins | 1.09 (0.12 - 10.01) | 0.940 | 4.79 (0.45 - 51.50) | 0.196 | 5.18 (0.72 - 37.45) | 0.103 |
| Number of lifetime sex partners | 1.34 (1.01 - 1.77) | 0.039 | 1.20 (0.84 - 1.71) | 0.325 | 0.95 (0.72 - 1.25) | 0.715 |
| Age of first sex | 1.03 (0.81 - 1.31) | 0.822 | 1.05 (0.81 - 1.38) | 0.701 | 1.01 (0.81 - 1.26) | 0.951 |

^1^IARC HR-HPV (IARC High-Risk HPV): HPV 16, 18, 31, 33, 35, 39, 45, 51, 52, 56, 58, 59, 66

^2^A9 HPV: HPV 16, 31, 33, 35, 52, 58

^3^Non-HPV 16 A9: HPV 31, 33, 35, 52, 58

^4^A7 HPV: HPV 18, 39, 45, 59, 68

^5^Non-HPV 18 A7: HPV 39, 45, 59, 68

^6^LR-HPV (Low-Risk HPV): HPV 6, 11, 40, 42, 54, 55, 61, 62, 64, 71, 72, 81, 83, 84, CP6108
